# Digitally Planned and Guide-Delivered Provisionalization for Emergence Profile Shaping in the Esthetic Zone: Clinical Outcomes and Complications in a Retrospective Single-Arm Cohort Study

**DOI:** 10.3390/jcm15103945

**Published:** 2026-05-20

**Authors:** Cristinel Adrian Nechita, Corina Marilena Cristache, Oana Elena Burlacu Vatamanu, Cristian Corneliu Butnarasu, Victor Nimigean

**Affiliations:** 1 Private Dental Practice (Oral Surgery), Newfast Implant, 625100 Adjud, Romania; drnechitacristinel@gmail.com; 2 Department of Dental Techniques, “Carol Davila” University of Medicine and Pharmacy, 8, Eroii Sanitari Blvd., 050474 Bucharest, Romania; 3 Doctoral School, “Carol Davila” University of Medicine and Pharmacy, 37 Dionisie Lupu Street, 020021 Bucharest, Romania; 4 Cad & Go Inception Dental Laboratory, 38 Delea Noua Street, 030925 Bucharest, Romania; cristian.butnarasu@gmail.com; 5 Deparment of Anatomy, Faculty of Dentistry, “Carol Davila” University of Medicine and Pharmacy, 8, Eroii Sanitari Blvd., 050474 Bucharest, Romania; victor.nimigean@umfcd.ro

**Keywords:** dental implants, single-tooth, dental prosthesis design, esthetics, dental, computer-aided design, dental abutments, immediate dental implant loading, dental implant-abutment design

## Abstract

**Background/Objectives:** Immediate provisionalization in the esthetic zone is a well-documented but technique-sensitive procedure, and the choice of provisional connection geometry, with or without an antirotational index, remains debated. The aim of this retrospective single-arm cohort clinical study was to evaluate the clinical performance of a digitally planned, guide-delivered provisionalization protocol using prefabricated provisional crowns connected to 5-degree Morse taper implants without an antirotational index, with emphasis on emergence profile shaping and peri-implant tissue stability at one year; **Methods:** Twenty consecutive single-implant cases treated according to the standardized protocol from January 2024 onward and completing at least one year of follow-up after definitive crown delivery by the February 2026 data-lock date were included (19 female, 1 male; mean age 38.1 ± 12.7 years; 18 anterior and 2 premolar sites). All implants were placed with primary insertion torque ≥ 30 N·cm (mean 34.75 ± 2.55 N·cm) and immediately restored with a digitally designed, non-antirotational provisional crown. Primary outcome was provisional retention without major intervention; secondary outcomes included biologic complications, papilla score, marginal bone change at T0–T3 and T3–T4, and buccal contour change (T0 vs. T2 intraoral scan superimposition). Wilson 95% confidence intervals, Fisher’s exact test, and Mann–Whitney U test were used (α = 0.05); **Results:** Provisional retention without major intervention was 75.0% (15/20; 95% CI 53.1–88.8). Biologic complications were uncommon (bleeding on probing, suppuration, midfacial recession, and chairside adjustment, each 5.0%). Mean total marginal bone loss at one year was 0.37 ± 0.20 mm; mean buccal contour gain was 1.41 ± 0.48 mm. A complete papilla was preserved in 70.0% of cases. **Conclusions:** Digitally planned, guide-delivered provisionalization on a non-antirotational 5-degree Morse taper interface appears clinically feasible for emergence profile shaping in the esthetic zone, with favorable peri-implant tissue outcomes at one year.

## 1. Introduction

Dental implant therapy in the esthetic zone increasingly emphasizes not only osseointegration and survival, but also the predictability of peri-implant soft-tissue architecture and the efficiency of restorative workflows. In single-tooth implant rehabilitation, the final esthetic outcome is strongly influenced by the contour, stability, and maturation of the peri-implant mucosa, particularly in the cervical and transmucosal regions [1]. Accordingly, immediate implant placement and immediate provisionalization have gained wide acceptance as strategies to reduce treatment time, limit the number of surgical interventions, support peri-implant soft-tissue healing, and guide the development of an individualized emergence profile [2,3]. Recent systematic reviews indicate that immediate implant placement in the esthetic zone can achieve high survival rates when strict case selection, adequate primary stability, prosthetically driven implant positioning, and careful management of hard and soft tissues are respected [4].

Implant placement in the esthetic zone remains clinically demanding because implant survival alone does not guarantee a successful treatment outcome. Esthetic success depends on the stability of peri-implant hard and soft tissues, maintenance of the midfacial mucosal margin, preservation of papillae, and creation of a natural emergence profile [1]. Following tooth extraction, dimensional changes of the alveolar ridge are expected, particularly at the facial aspect, and these changes may compromise the esthetic result if they are not anticipated during implant planning and provisional restoration design [5]. Therefore, immediate provisionalization should be considered not merely as a temporary restorative phase, but as an integral component of peri-implant tissue management.

In immediate implant protocols, the provisional restoration functions as both a prosthetic and biological device. Its cervical and transmucosal contours may support the pre-existing gingival architecture, protect the blood clot and grafting material, and guide soft-tissue maturation during healing [6]. The concept of contour management has been refined by distinguishing the critical contour, which mainly influences the gingival margin level and zenith, from the subcritical contour, which affects soft-tissue support, gingival color, and the available regenerative space [7]. For immediate implant restorations, the critical contour should generally support the existing marginal tissue architecture, whereas the subcritical contour may be designed with a concave configuration to avoid excessive tissue compression and to provide space for healing, graft maturation, and soft-tissue thickening [8].

More recently, the emergence profile has also been described through the Esthetic Biological Contour framework, which divides the transmucosal restorative complex into esthetic, bounded, and crestal zones. This model emphasizes that emergence profile design is influenced by the three-dimensional position of the implant, the available soft-tissue volume, the restorative material, and the morphology and surface characteristics of the transmucosal component. From this perspective, the provisional restoration delivered at the time of implant placement may play a decisive role in establishing a biologically compatible and esthetically favorable peri-implant contour [8].

Computer-guided implant surgery was introduced to improve the transfer of a prosthetically driven virtual plan to the clinical situation [9]. This approach is particularly useful for immediate implant placement in the esthetic zone, where the buccolingual, mesiodistal, and apicocoronal position of the implant strongly influences the restorative emergence profile and the risk of midfacial recession [10]. However, guided surgery does not completely eliminate clinical deviations [11]. Linear and angular discrepancies between the planned and placed implant positions are still reported in the literature, and these deviations may become clinically relevant when a prefabricated provisional restoration is intended to be inserted immediately after implant placement [12].

This issue is particularly relevant when a prefabricated screw-retained provisional crown is manufactured with an antirotational index, as is typically recommended for single implant restorations. Antirotational components are designed to ensure a unique rotational position of the restoration and to prevent prosthetic rotation [13]. Nevertheless, when the provisional crown is digitally designed before surgery and delivered immediately after guided implant placement, even minor deviations in implant position, angulation, or depth may prevent the planned emergence profile and facial contour from seating in the most favorable clinical orientation. In this specific context, a non-antirotational provisional design may offer a practical advantage by allowing intraoperative rotational adaptation of the crown until the cervical contour is optimally aligned with the peri-implant soft-tissue architecture.

The use of a Morse taper implant-abutment connection may further support this approach from a mechanical and biological standpoint. Conical implant-abutment connections have been associated with improved abutment fit, reduced microgap formation, greater resistance to abutment micromovement, favorable torque maintenance, and improved seal performance compared with several non-conical connection designs [14]. Schmitt et al. reported that conical connections generally demonstrated superior performance in terms of abutment fit, stability, and seal, while clinical studies showed comparable implant survival rates and, in several cases, reduced marginal bone loss around conical connection implants [15]. Although no implant-abutment connection provides a complete bacterial seal, conical connections appear to reduce bacterial contamination and microgap enlargement under loading. In addition, loading may induce a frictional locking effect between the implant and abutment, sometimes described as cold welding; however, this phenomenon is system-dependent and influenced by torque, loading conditions, and component design, and therefore should not be overinterpreted clinically [16].

Although the available literature supports immediate implant placement and immediate provisionalization in carefully selected esthetic cases, limited evidence is available regarding prefabricated, guide-delivered provisional crowns connected through a non-antirotational Morse taper interface. This clinical concept differs from conventional single-crown protocols, in which an antirotational connection is typically preferred. The potential advantage of the non-antirotational design is that it may compensate for minor discrepancies between the planned and actual implant position by allowing rotational adaptation of the provisional emergence profile at the time of surgery. Therefore, the present retrospective study was designed to evaluate the clinical outcomes and biological and technical complications of digitally planned and guide-delivered emergence profile shaping using a screw-retained composite provisional crown connected to a 5-degree Morse taper implant without an antirotational index, in the esthetic zone.

The primary null hypothesis was that this digitally planned, guide-delivered non-antirotational provisionalization protocol would not demonstrate preliminary clinical feasibility, defined as provisional survival without major intervention in most treated cases until definitive crown delivery. The secondary null hypotheses were that the protocol would not be associated with favorable peri-implant tissue outcomes, including low biologic complication rates, absence of clinically relevant midfacial mucosal recession, preservation of papilla fill, limited marginal bone-level change, and positive buccal contour change during the tissue-conditioning phase. Because no indexed-control or conventional provisionalization group was included, these hypotheses were not intended to test comparative superiority or to attribute the observed outcomes specifically to the absence of an antirotational index.

## 2. Materials and Methods

### 2.1. Study Design and Ethical Approval

This retrospective single-arm clinical study was conducted in a dental clinic affiliated with “Carol Davila” University of Medicine and Pharmacy, Bucharest, Romania and reported in accordance with Strengthening the Reporting of Observational Studies in Epidemiology statement for observational studies (STROBE) and the check list is provided as Supplementary Materials. Consecutive patients treated according to the standardized clinical and digital protocol from January 2024 onward were screened for eligibility. No formal a priori sample-size calculation was performed. The final sample was determined by the number of eligible consecutive patients who fulfilled all inclusion criteria, met none of the exclusion criteria, and had completed at least one year of follow-up after definitive crown delivery by the data-lock date in February 2026.

The study was conducted in accordance with the ethical principles of the World Medical Association Declaration of Helsinki, the Belmont Report, the Council for International Organizations of Medical Sciences guidelines, and the International Council for Harmonisation Good Clinical Practice guidelines. Ethical approval was obtained from the Ethics Committee of “Carol Davila” University of Medicine and Pharmacy, Bucharest, Romania (approval number 36368/2023). Written informed consent was obtained from all patients for the clinical procedures and use of anonymized clinical data. All surgical and prosthetic procedures were performed by the same experienced implantologist, C.A.N.

### 2.2. Case Selection

#### Inclusion and Exclusion Criteria

Patients were screened according to predefined inclusion and exclusion criteria. Only consecutive cases that fulfilled all inclusion criteria and none of the exclusion criteria were included in the final analysis. The eligibility criteria are summarized in Table 1.

### 2.3. Digital Planning Protocol

After the initial clinical examination, intraoral digital impressions of the arch to be restored, the antagonist arch, and the centric occlusion record were obtained using a Carestream 3600 intraoral scanner (Carestream Dental LLC, Atlanta, GA, USA). Scan acquisition, processing, and export were performed using the latest version of DEXIS IS ScanFlow software, version 1.0.11 (Quakertown, PA, USA). The resulting STL files were exported for digital planning.

A Cone Beam Computed Tomography (CBCT) scan was performed for each patient using a NewTom VGi evo unit (NewTom, Imola, Bologna, Italy). STL and DICOM files were imported into R2GATE software, version 2.20 (MegaGen, Daegu, Republic of Korea), and merged using the best-fit repositioning tool. Manual fine adjustments were performed when necessary [17].

A prosthetically driven digital wax-up was designed according to functional and esthetic requirements. Implant position, diameter, and length were planned based on the final restorative design, the available bone volume, bone quality, and anatomical landmarks. After review and approval of the digital treatment plan by the clinician, the provisional crown was designed in Exocad^®^ DentalCAD, version 3.2 Elefsina software (Exocad GmbH, Darmstadt, Germany), according to the Esthetic Biological Contour Concept 2.0 [8]. The design approach is also consistent with the dynamic compression technique proposed by Wittneben et al., in which provisional restorations are used to actively condition peri-implant soft tissues through controlled selective pressure, followed by strategic reduction or under-contouring to create space for papillary and sulcular tissue fill and maturation [18].

The surgical guide was designed in R2Ware, version 1.10820 (MegaGen, Daegu, Republic of Korea), without metallic sleeve, corresponding to the guided surgical protocol as described in previously published papers [17,19,20].

### 2.4. Surgical Guide and Provisional Fabrication

Surgical guides were 3D printed using NextDent SG resin (Vertex-Dental B.V., Soesterberg, The Netherlands) on a Phrozen Sonic XL 4K printer (3Dream Teknoloji, Istanbul, Turkey), based on Digital Light Processing technology. The use of the Phrozen printer in clinical digital workflows has been supported by dimensional-verification data. Beidas et al. reported reproducible results for devices printed with a Phrozen 4K printer, with an average root mean square (RMS) deviation of approximately 0.20 mm after correction for processing and analysis error [21]. In the present study, after printing, all surgical guides were cleaned and post-cured according to the applied laboratory protocol and the manufacturer’s instructions. When applicable, working models incorporating virtual extractions were printed separately using a standard model resin. The guides were inspected on the corresponding printed models and clinically verified for complete seating and stability before surgery.

The provisional restorations were fabricated using non-hexagonal ZrGEN titanium bases corresponding to the planned implant diameter, gingival height, and post height. The provisional crowns were milled from Ruthinium PMMA discs (Ruthinium—Dental Manufacturing S.p.A., Badia Polesine, Rovigo, Italy) and cemented on the corresponding Ti bases.

### 2.5. Surgical and Provisionalization Protocol

Before surgery, all patients rinsed with 0.2% chlorhexidine for 1 min to reduce bacterial contamination. Local anesthesia was administered using 4% articaine with epinephrine 1:100,000 by buccal and palatal infiltration. When extraction was required, the failing tooth was removed atraumatically using periotomes and forceps, with care taken to avoid pressure on the buccal plate. The socket was thoroughly debrided under sterile saline irrigation, and the integrity of the socket walls was assessed visually and with a periodontal probe.

A flapless approach was used whenever possible. A minimal flap was elevated only when required to facilitate adjunctive grafting or defect management. Implant placement was performed through a patient-specific stereolithographic surgical guide using a torque-controlled handpiece under copious sterile saline irrigation, according to the manufacturer’s instructions. All implants were MegaGen AnyRidge implants (MegaGen, Daegu, Republic of Korea) featuring a 5-degree internal Morse taper connection and platform switching, selected according to the digital treatment plan. The implant platform was positioned approximately 3 mm apical to the planned mucosal zenith and at least 2 mm palatal to the buccal cortical plate. Final insertion torque was recorded.

When a peri-implant gap larger than 2 mm was present between the implant surface and the buccal plate, or when buccal dehiscence required contour augmentation, the defect was grafted with a slow-resorbing deproteinized bovine bone mineral xenograft. When a thin tissue phenotype or additional buccal contour gain was indicated, a subepithelial connective tissue graft (SCTG) was harvested from the lateral palate, introduced into a supraperiosteal envelope through an intrasulcular tunnel approach, and stabilized with resorbable sutures.

Immediately after implant insertion, the prefabricated provisional crown, digitally designed before surgery, was seated on a non-antirotational titanium base. The absence of an antirotational index allowed fine rotational adjustment of the crown until its cervical and facial contours were aligned with the planned peri-implant soft-tissue architecture. The temporary crown screw was then tightened to 15 N·cm using a calibrated torque wrench.

Occlusion was assessed in maximum intercuspation and during protrusive and lateral excursions using articulating paper, and the provisional was adjusted to achieve light or no contact in maximum intercuspation and complete absence of contact during eccentric movements.

Chairside adjustments at the time of delivery were recorded as absent, minor, or major. Minor adjustments included polishing, proximal contact correction, or occlusal adjustment. Major intervention was defined as provisional remake, relining beyond minor polishing, or conversion to another provisional retention concept.

All patients received postoperative antibiotic therapy consisting of amoxicillin 875 mg with clavulanic acid 125 mg twice daily for 5 days.

### 2.6. Follow-Up Schedule and Data Collection

Patients were evaluated at the following time points:

T0: Surgical day, including implant placement and provisional restoration delivery. A postoperative orthopantomogram (OPT) was obtained.

T1: 1–2 weeks postoperatively, for clinical evaluation and occlusal adjustments when required.

T2: Tissue maturation visit and digital impression for the definitive crown, generally 4–8 months after surgery. An intraoral scan (IOS) was performed for definitive crown fabrication. Both intraoral scans used for buccal contour evaluation—the baseline scan before implant insertion and the scan obtained after emergence-profile conditioning at the prosthetic phase (T2)—were acquired with the same scanner and software workflow to minimize inter-scanner variability during scan superimposition.

T3: Definitive crown delivery. A control OPT was obtained.

T4: 12 months after definitive crown delivery. A follow-up OPT was obtained.

Additional visits were scheduled and recorded, when necessary, particularly in cases of mechanical or clinical complications.

### 2.7. Data Extraction

Data were extracted from clinical records, digital planning files, intraoral scans, photographs, and radiographs, when available. Before final data extraction, two reviewers (C.C.B. and O.E.B.V.) were calibrated on five randomly selected cases to standardize the extraction process and all measurement procedures, including marginal bone-level measurements, papilla scoring, and buccal contour assessment based on intraoral scan superimposition. After calibration, the two reviewers independently extracted the data and performed the measurements using the same predefined protocol. Disagreements were resolved by consensus under the supervision of a third reviewer (C.M.C.).

To address potential sources of bias, only cases treated according to the same standardized surgical and prosthetic protocol by a single experienced operator were included. Consecutive screening of all eligible patients was performed according to predefined inclusion and exclusion criteria to reduce selection bias. Primary and secondary outcomes were defined a priori and assessed using structured case-report forms based on objective clinical, digital, and radiographic measurements. Independent data extraction and duplicate measurements by calibrated reviewers were used to reduce information and measurement bias. Nevertheless, because of the retrospective single-arm design, the absence of randomization, and the lack of a control group, residual selection, reporting, and confounding bias cannot be excluded.

The following patient-level variables were recorded: age, sex, smoking status, periodontal history, systemic conditions relevant to healing, and bruxism or other parafunctional habits.

Site- and restoration-related variables included tooth position, tissue phenotype, socket status, surgical approach, and hard- or soft-tissue grafting procedures.

Surgical variables included implant diameter and length, insertion torque, and intraoperative deviations, if present.

Provisional restoration variables included the occlusal scheme, chairside adjustments at delivery, and complications related to the provisional restoration.

**Figure 1:**
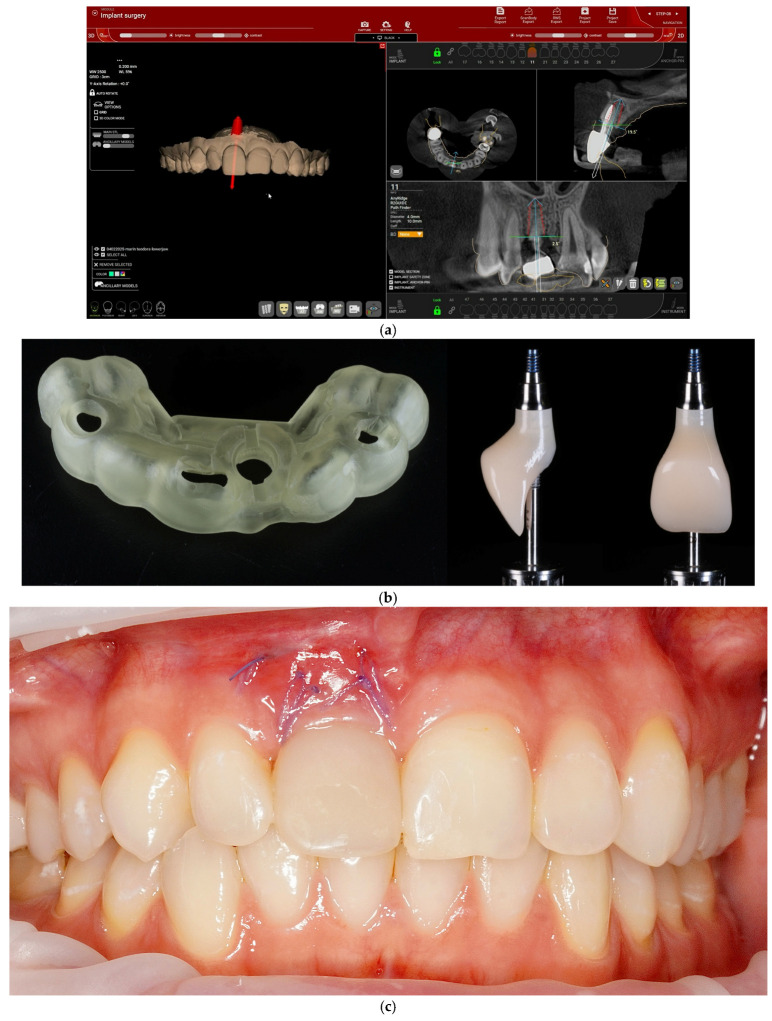

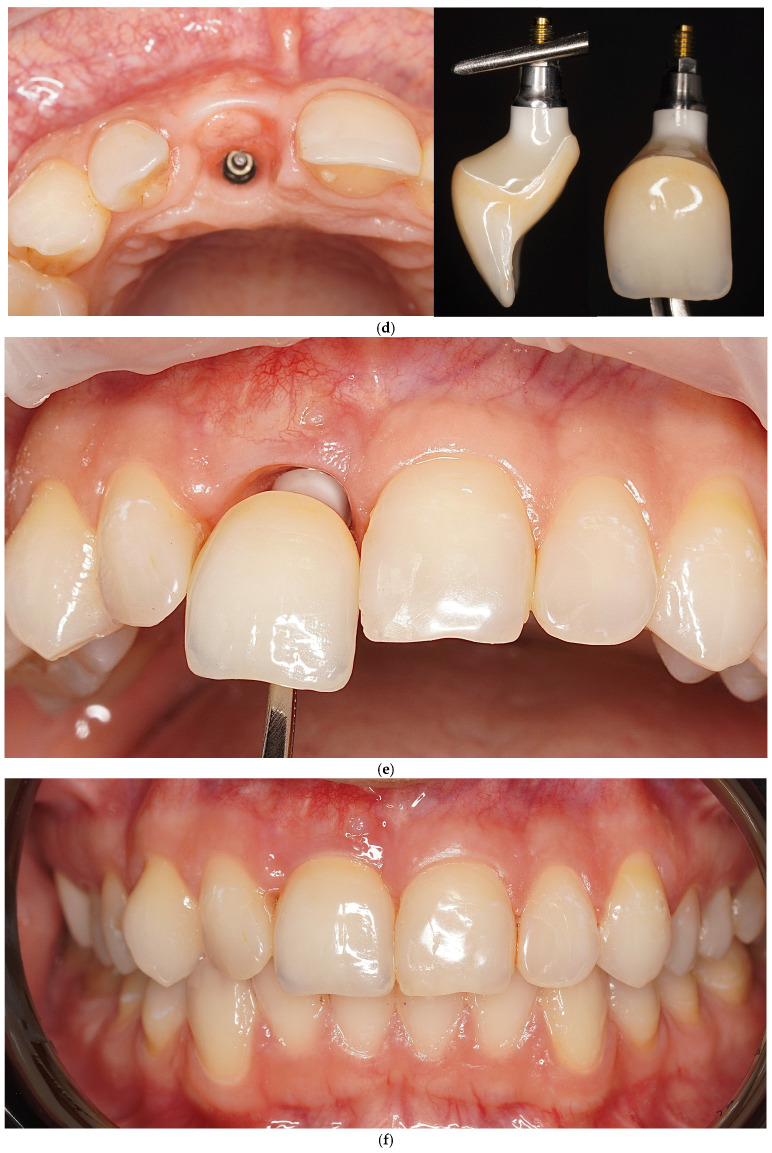
Digital workflow and clinical sequence for immediate implant placement and immediate provisional restoration of tooth no. 11. (**a**) Implant planning in R2Gate software based on digital data integration. (**b**) 3D-printed surgical guide and customized provisional crown prepared for delivery at the time of implant placement. (**c**) Provisional restoration inserted immediately after tooth extraction, implant placement, bone and soft-tissue grafting. (**d**) Mature peri-implant soft tissues and emergence profile at the definitive restoration stage, together with the definitive zirconia crown. (**e**) Delivery of the definitive crown. (**f**) Clinical aspect at the 1-year follow-up visit after definitive crown delivery (T4).

The workflow for one of the 20 included cases is illustrated in Figure 1.

### 2.8. Outcome Measures

The primary outcome was provisional survival without major intervention until definitive crown delivery. This was defined as the provisional restoration remaining clinically functional without dislodgement requiring a new retention method, remake or relining beyond minor polishing, or conversion to an indexed antirotational titanium base or alternative provisional approach.

Secondary outcomes included mechanical, biologic, esthetic, and process-related outcomes.

Mechanical complications included provisional dislodgement, loss of retention, rotation events, fracture or chipping, screw loosening where applicable, and unplanned conversion to another provisional method.

Biologic and esthetic outcomes included midfacial mucosal recession, papilla score, bleeding on probing, suppuration, peri-implant mucositis diagnosis, and marginal bone-level change where documented.

Process outcomes included the number of unplanned visits related to the provisional restoration and the need for chairside adjustment.

Marginal bone levels were assessed on OPTs obtained at T0, T2, and T4 using ImageJ software version1.54p (https://ij.imjoy.io/; accessed on 30 April 2026). Each OPT was calibrated individually using the known implant length to correct for image magnification. For each implant, linear measurements were performed on the mesial and distal aspects from the implant shoulder to the first visible bone-to-implant contact. Measurements were recorded in millimeters, and the mean marginal bone level for each implant was calculated as the average of the mesial and distal values.

Marginal bone-level changes were calculated by comparing measurements between time points. Early marginal bone change was defined as the difference between T0 and T3, whereas late marginal bone change was defined as the difference between T3 and T4. A positive value indicated apical displacement of the bone level relative to the implant shoulder, corresponding to marginal bone loss, whereas a negative value indicated coronal bone gain. Mesial and distal changes were first calculated separately and then averaged to obtain the mean marginal bone-level change per implant.

**Figure 2:**
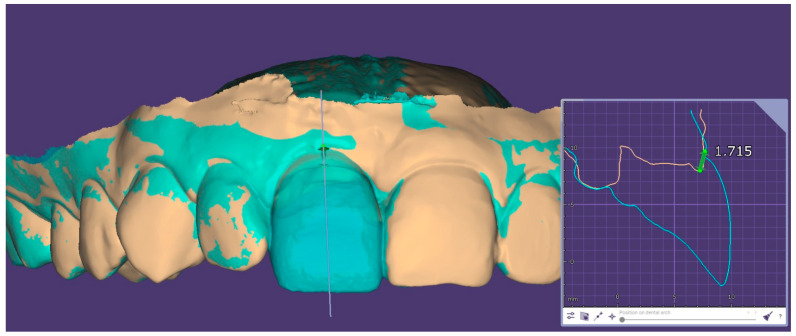
Digital superimposition in Exocad of the preoperative intraoral scan (T0, beige) and the intraoral scan acquired at the prosthetic conversion visit (T2, cyan), aligned by surface-based best-fit registration on the adjacent teeth. The vertical pink line indicates the reconstructed implant long axis. The right inset displays the sagittal cross-section perpendicular to the dental arch passing through the mid-buccal point of the implant, showing the T0 (orange) and T2 (cyan) mucosal contours on a millimetric grid; the green double-arrow represents the linear bucco-palatal distance between the two contours at the mid-buccal level, here measuring 1.72 mm of buccal contour gain induced by the provisional restoration.

At the prosthetic conversion visit (T2), corresponding to the tissue maturation stage before definitive restoration, the provisional crown was removed, and a new intraoral scan was performed to record the conditioned peri-implant mucosal architecture and emergence profile. The T2 intraoral scan was imported into Exocad version 3.2 Elefsina and superimposed with the initial preoperative intraoral scan obtained before implant placement, using a surface-based best-fit registration anchored on the occlusal and palatal surfaces of the adjacent teeth, while the peri-implant region was excluded from the alignment mask to avoid bias. After registration, the implant long axis was reconstructed and a sagittal cross-section was generated perpendicular to the dental arch, passing through the mid-buccal point of the implant. On this cross-section, three horizontal reference lines were drawn perpendicular to the implant axis, at the level of the mucosal margin, 1 mm apical, and 2 mm apical to it. At each level, the linear bucco-palatal distance between the baseline (T0) and the T2 mucosal contours was measured in millimeters, with positive values indicating buccal contour gain and negative values indicating buccal collapse; the mean of the three readings was recorded as the buccal contour change per implant (Figure 2). This comparison between the baseline IOS and the T2 IOS was used for data collection and quantitative assessment of soft-tissue contour changes induced by the provisional restoration. Similar intraoral scan-based or STL superimposition approaches have been used in previous studies to evaluate peri-implant mucosal margin changes, soft-tissue thickness, buccal contour alterations, and volumetric tissue changes around implant sites [22,23,24].

The mesial and distal papillae were evaluated using the papilla index described by Jemt [25]. A score of 0 indicated absence of the papilla; 1, less than half of the papilla height present; 2, at least half of the papilla height present but incomplete fill of the interproximal space; 3, complete papilla fill; and 4, hyperplastic papilla tissue. Mesial and distal papillae were scored separately on standardized intraoral photographs obtained at T2 and T4. For each implant site, the less favorable score between the mesial and distal papillae was recorded for analysis.

### 2.9. Statistical Analysis

Statistical analysis was performed using JASP software (version 0.95.4.0 for Windows). No formal a priori sample-size calculation was performed. Because this was a single-arm retrospective case series with a limited sample size, the analysis was primarily descriptive. Continuous variables (age, number of unplanned visits, insertion torque, implant diameter, implant length) were summarized as mean, standard deviation, median, interquartile range, minimum and maximum. Categorical variables (sex, smoking status, periodontal history, systemic conditions, tooth position, tissue phenotype, socket status, flap design, grafting procedure, occlusal scheme, chairside adjustments, dislodgement, midfacial mucosal recession, papilla score, bleeding on probing, suppuration, and marginal bone-level change) were reported as frequencies and percentages. The primary outcome—provisional survival without major intervention—and all binary secondary outcomes were reported as proportions with 95% Wilson confidence intervals. For exploratory hypothesis-generating analyses, associations between provisional dislodgement and categorical predictors (smoking status, periodontal history, tissue phenotype, socket status, grafting, occlusal scheme, tooth-position group, flap design, systemic conditions, and insertion-torque category) were assessed using Fisher’s exact test (2 × 2) or the Freeman–Halton extension (r × c). Continuous and ordinal variables (number of unplanned visits, papilla score, age, insertion torque) were compared between cases with and without dislodgement using the Mann–Whitney U test. A two-sided α = 0.05 was used; given the exploratory nature of the secondary analyses, no adjustment for multiple comparisons was applied, and significant findings are reported as hypothesis-generating only.

## 3. Results

Twenty consecutive patients treated according to the standardized protocol from January 2024 onward met the inclusion criteria, each contributing one implant in the esthetic zone, and all had completed at least one year of follow-up after definitive crown delivery by the February 2026 data-lock date. Mean age was 38.1 ± 12.7 years (median 36; range 25–67). Smoking status was negative in 14 patients (70.0%); periodontal history was gingivitis in 13 (65.0%), chronic periodontitis in 6 (30.0%), and unremarkable in 1 (5.0%); and 18 patients (90.0%) reported no systemic conditions, with 2 (10.0%) presenting controlled hypertension. None of the patients were diagnosed with bruxism or other parafunctional habits. The implants were placed in 18 anterior tooth positions (90.0%) and in 2 premolar sites (10.0%). Tissue phenotype was thin in 12 cases (60.0%), mixed in 4 (20.0%), and thick in 4 (20.0%). Thirteen sites (65.0%) presented healed sockets, while 7 (35.0%) underwent immediate placement into post-extraction sockets. A flapless approach was used in 19 cases (95.0%) and a one-sided minimal flap in a single case (5.0%). Soft-tissue grafting alone was performed in 10 cases (50.0%), combined soft- and hard-tissue grafting in 8 (40.0%), and no grafting in 2 (10.0%). All implants achieved primary stability adequate for immediate provisionalization, with mean insertion torque of 34.75 ± 2.55 N·cm (range 30–40); 15 implants (75.0%) were placed at 35 N·cm, 3 (15.0%) at 30 N·cm, and 2 (10.0%) at 40 N·cm. Implant diameters were 3.5 mm in 11 cases (55.0%) and 4.0 mm in 9 (45.0%), with implant length ranging from 10 to 13 mm (mean 11.8 ± 1.0). All 20 implants integrated successfully and supported the planned definitive crown. The sample characteristics of the included participants are shown in Table 2.

No implant loss was registered during the entire follow-up period. The primary outcome was provisional survival without major intervention. No case required a remake, conversion to a titanium base with an antirotational index, or alternative provisional approach. The provisional restoration was retained in place without dislodgement throughout the maturation phase in 15 of 20 cases (75.0%; Wilson 95% CI 53.1–88.8%). Provisional dislodgement occurred in the remaining 5 cases (25.0%; 95% CI 11.2–46.9%); all dislodgement events were managed chairside by screwing or re-cementation, with no damage to the implant–abutment connection, no loss of soft-tissue conditioning, and no compromise of the planned definitive restoration. Definitive crown delivery was achieved in 100% of cases.

Mechanical and biologic complications were uncommon (Table 3). Chairside occlusal adjustment at delivery was required in 1 case (5.0%; 95% CI 0.9–23.6%). Bleeding on probing was present in 1 case (5.0%), suppuration in 1 case (5.0%), and any midfacial mucosal recession (1 mm) in 1 case (5.0%). The recession was managed successfully with soft-tissue grafting. A complete papilla (papilla score = 3) was preserved in 14 of 20 cases (70.0%; 95% CI 48.1–85.5%); the remaining 6 cases (30.0%) presented partial papilla fill (score = 2). The number of unplanned visits during the maturation phase ranged from 0 to 4 (mean 1.0 ± 1.4; median 0 [IQR 0–2]); 12 patients (60.0%) had no unplanned visits, while 8 (40.0%) required at least one unplanned visit, predominantly for screwing (*n* = 4) or re-cementation (*n* = 1) of a dislodged provisional.

Quantitative marginal bone-level change was measured on OPTs at the implant placement (T0), at definitive crown delivery (T3), and at the end of follow-up (T4, 12 months). Mean early bone change between T0 and T3 was 0.24 ± 0.20 mm (median 0.20; range 0.02–0.84), while mean late bone change between T3 and T4 was 0.14 ± 0.03 mm (median 0.13; range 0.10–0.18). Cumulative mean total bone loss at 12 months was 0.37 ± 0.20 mm (median 0.33; range 0.14–0.96). Late bone change was numerically smaller than early change in 11 of 20 cases and approximately equal in the remaining cases (Wilcoxon signed-rank W = 53.5, *p* = 0.054), indicating that most of the peri-implant bone remodeling occurred during the early healing and tissue-conditioning phase, with limited radiographic marginal bone-level change after delivery of the definitive crown, within the limitations of panoramic imaging. Total bone loss remained below the 0.5 mm threshold in 16 of 20 cases (80.0%; 95% CI 58.4–91.9%) and below the 1.0 mm threshold in all 20 cases (100.0%; 95% CI 83.9–100.0%). Only one case (5.0%) crossed the categorical threshold of >0.5 mm to fall in the <1 mm class. No case showed total bone loss greater than 1.0 mm at one year (Table 4).

The change in facial peri-implant mucosal contour, measured by superimposing intraoral scans at baseline (T0) and after soft-tissue conditioning (T2), was positive (gain) in all 20 cases. Mean buccal contour gain was 1.41 ± 0.48 mm (median 1.44; range 0.68–2.29). Categorical assessment classified 5 cases (25.0%) as mild gain (<1.0 mm), 12 cases (60.0%) as moderate gain (1.0–1.9 mm), and 3 cases (15.0%) as pronounced gain (≥2.0 mm). No case showed a negative buccal contour change. However, because most sites received soft- and/or hard-tissue grafting, the observed buccal contour gain should be interpreted as the combined result of the complete clinical protocol, including provisional contour design, grafting, socket healing, tissue phenotype, and guided implant placement, and not as an isolated effect of the provisional restoration (Table 5).

### Exploratory Associations with Provisional Dislodgement

Given the limited sample size and the small number of dislodgement events, all subgroup and association analyses were interpreted as descriptive and hypothesis-generating only.

Categorical predictors of provisional dislodgement were tested using Fisher’s exact test (2 × 2) or the Freeman–Halton extension (r × c). None of the predictors examined reached statistical significance: smoking (*p* = 1.000), periodontal history (*p* = 0.255), tissue phenotype (*p* = 0.092), socket status (*p* = 0.290), grafting protocol (*p* = 0.230), occlusal scheme (*p* = 0.645), tooth position (*p* = 1.000), flapless approach (*p* = 0.250), systemic conditions (*p* = 1.000), and insertion-torque category (*p* = 0.730). Continuous and ordinal variables were compared between dislodged and retained cases using the Mann–Whitney U test. The number of unplanned visits was significantly higher in dislodged cases (median 2 [IQR 2–4] vs. 0 [IQR 0–1]; U = 61.5, *p* = 0.019), but this association is mechanistic (each dislodgement triggers a screwing or re-cementation appointment) and does not represent an independent predictor. No statistically significant differences between dislodged and retained cases were found for papilla score (*p* = 0.111), age (*p* = 0.220), insertion torque (*p* = 0.818), early bone loss (*p* = 0.406), late bone loss (*p* = 1.000), total bone loss (*p* = 0.407), or buccal contour gain (*p* = 0.793). Spearman correlations among continuous outcomes were also non-significant: total bone loss did not correlate with insertion torque (ρ = −0.16, *p* = 0.51), with buccal contour gain (ρ = −0.33, *p* = 0.15), or with age (ρ = 0.07, *p* = 0.76) (Table 6).

## 4. Discussion

The present retrospective single-arm clinical study evaluated a digitally planned and guide-delivered provisionalization protocol for emergence profile shaping in the esthetic zone using prefabricated provisional crowns connected to 5-degree Morse taper implants without an antirotational index. The distinctive feature of this protocol is the intentional use of a non-antirotational provisional component to allow intraoperative rotational adaptation of the provisional crown after guided implant placement. This differs from conventional single-implant provisionalization, in which an antirotational internal geometry, such as an internal hex, is typically used to restrict rotational freedom and guide the provisional restoration toward a reproducible prosthetic position. In the present workflow, the absence of an index was not intended as a mechanical simplification alone, but as a clinically useful feature that allows the clinician to rotate the provisional until the cervical and facial contours are aligned with the planned peri-implant soft-tissue architecture.

The null hypotheses were interpreted descriptively because of the retrospective single-arm design and limited sample size. The primary null hypothesis was not supported, as provisional survival without major intervention was achieved in 15 of 20 cases; however, the 25% dislodgement rate shows that the protocol was not free of complications and should be regarded as feasible, but not predictably uncomplicated. For the secondary outcomes, the null hypotheses were not supported descriptively, as biologic complications were uncommon, midfacial mucosal recession was rare, papilla fill was generally preserved, marginal bone-level change was limited, and buccal contour gain was positive in all cases. However, these outcomes cannot be attributed specifically to the non-antirotational connection because no indexed-control group was included and most cases received hard- and/or soft-tissue grafting. For the exploratory analyses, no relevant predictors were identified, except for the expected association between dislodgement and unplanned visits. Given the small sample and limited number of events, these findings should be considered hypothesis-generating only.

Immediate implant placement and immediate provisionalization in the esthetic zone are well documented but remain technique-sensitive [4]. A recent systematic review and meta-analysis including 63 studies reported 1-year implant survival of 99.2% and 1-year restoration survival of 98.9% for immediately placed and immediately loaded single implants in the esthetic zone, with generally favorable esthetic outcomes up to 5 years [4]. These findings support the biological plausibility of the present protocol, provided that strict case selection, prosthetically driven implant positioning, primary stability, and careful occlusal control are respected. The present study adds to this literature by focusing on a specific restorative variable that has received limited attention: the use of a guide-delivered, prefabricated provisional crown without an antirotational index on a Morse taper implant–abutment connection.

The biological rationale for the protocol is consistent with current concepts of emergence profile management. The provisional restoration is not merely a temporary crown but a tissue-conditioning device. It supports the peri-implant mucosa, protects the healing socket or grafted region, and guides maturation of the cervical architecture. The Esthetic Biological Contour concept emphasizes that the morphology, material, and surface characteristics of the transmucosal component influence esthetic and biological tissue behavior [8]. In particular, concave or under-contoured subgingival designs have been associated with more stable mucosal margins and reduced tissue compression [8]. This is relevant to the present study because the provisional crowns were digitally designed according to the planned emergence profile and then clinically positioned to optimize soft-tissue support.

Most peri-implant marginal bone remodeling in the esthetic zone occurs during the first 3–6 months after implant placement and is concentrated at the buccal aspect of the alveolar crest, where bundle bone undergoes resorption following tooth extraction [26,27]. Recent meta-analytic data on immediately placed and immediately loaded single implants in the esthetic zone report mean mesial and distal bone-level changes well below 1 mm at 12 months across most reconstructive protocols [4]. This is consistent with the cumulative bone loss of 0.37 ± 0.20 mm observed in the present cohort and with the stability observed during the late healing phase. Marginal bone-level changes were assessed on calibrated orthopantomograms, which were part of the routine clinical follow-up protocol used for implant patients in this clinic. Although calibration by known implant length was performed to reduce magnification-related error, panoramic radiographs are less precise than standardized periapical radiographs with individualized holders or CBCT-based protocols for detecting small peri-implant bone-level changes. Therefore, small mean changes such as those observed in the present study may fall within the measurement limitations of panoramic imaging. Postoperative or follow-up CBCT was not routinely performed in uncomplicated cases because additional three-dimensional imaging was not clinically justified and would conflict with radiation-protection principles, including ALARA [28,29]. This approach is consistent with implant-imaging recommendations indicating that CBCT should not be used for routine periodic assessment of asymptomatic implants and should be reserved for selected cases with clinical signs, symptoms, or suspected complications. Consequently, the marginal bone-level results should be interpreted descriptively as limited radiographic changes on calibrated panoramic images, not as precise evidence of bone stability at the submillimeter level.

The use of a non-antirotational component represents the most distinctive aspect of this workflow. In static guided surgery, minor deviations between the planned and final implant position may still occur, and these deviations become clinically relevant when a prefabricated provisional crown has a fixed antirotational index, because the crown can then seat only in one rotational position. A non-antirotational component allows the clinician to compensate for such deviations by rotating the provisional crown into the most favourable cervical and facial orientation, which is particularly advantageous in the esthetic zone, where small discrepancies in contour, contact position, or facial emergence can influence the mucosal margin and papillary support.

The mechanical feasibility of this approach depends largely on the Morse taper connection. Conical implant–abutment interfaces have been associated with improved abutment fit, reduced microgap formation, better seal performance, greater torque maintenance, and improved abutment stability compared with non-conical designs, and clinical studies have reported comparable survival rates and, in several cases, less marginal bone loss around conical-connection implants [14,15]. These characteristics support the use of a 5-degree Morse taper as a stabilizing mechanism for a provisional restoration without a conventional antirotational index [30]. Manufacturer-recommended abutment screw torque values for conical connections (typically 25–35 N·cm) are required to fully seat the interface and to develop the frictional locking effect that minimizes microgap formation and abutment micromovement [15,31]. During the provisional phase, however, a reduced screw torque (15–20 N·cm) is often applied to titanium-base components to facilitate eventual disassembly and chairside management without compromising the implant–abutment seal [32,33].

The intimate contact between conical metallic surfaces under seating torque and functional loading may produce a frictional locking effect, often described as “cold welding”. This effect is system-dependent and influenced by taper angle, seating force, screw torque, surface characteristics, and loading conditions, and the term should therefore be used cautiously: the 5-degree Morse taper likely contributed to provisional retention in our cohort through frictional engagement, but the results should not be interpreted as proof of a true metallurgical weld. A recent scoping review further reported that prosthetic index structures in Morse taper systems may improve positioning accuracy but can also influence stress distribution, reverse torque values, and biomechanical stability, and may modify or reduce the frictional engagement normally observed in conical connections [14]. This supports the conceptual basis of the present study—namely, that avoiding an antirotational index may preserve the frictional Morse taper engagement while allowing rotational adaptation of the provisional crown—although non-antirotational components may also increase the risk of mispositioning or microleakage if not seated carefully, so this approach should be considered a controlled clinical protocol rather than a universal recommendation.

Current consensus identifies a primary implants insertion torque of ≥32–35 N·cm as the threshold below which immediate provisionalization should be approached with caution, since lower values are associated with greater micromotion and increased risk of early failure [34,35]. In our cohort, all 20 implants achieved an insertion torque ≥ 30 N·cm and 85% reached ≥35 N·cm, which is in line with the threshold recommended by the International Team for Implantology (ITI) Consensus for type 1A protocols [36].

In the present study, provisional dislodgement occurred in 5 of 20 cases, while 15 cases remained free of dislodgement. Therefore, the findings should not be interpreted as indicating an uncomplicated protocol. Instead, they suggest that most provisionals remained clinically stable until definitive restoration, while a clinically relevant minority required chairside intervention. Although these events did not compromise the definitive restoration, they increased the need for unplanned maintenance visits and should be considered part of the clinical burden of the technique. The only statistically significant exploratory finding was that cases with provisional dislodgement required more unplanned visits than cases without dislodgement. Other variables, including smoking, periodontal history, tissue phenotype, socket status, grafting, occlusal scheme, tooth position, flap design, systemic conditions, and insertion torque category, were not significantly associated with dislodgement in this small sample. This indicates that dislodgement was the main driver of additional maintenance visits, but no reliable predictor could be identified. No reliable predictors of provisional dislodgement could be identified in this cohort. However, this should not be interpreted as evidence that patient-, site-, surgical-, prosthetic-, or occlusal-related variables are unimportant. The study included only 20 cases and five dislodgement events; therefore, the exploratory analyses were underpowered and should be considered descriptive and hypothesis-generating only. Larger controlled studies are needed to evaluate potential predictors of dislodgement and maintenance burden.

Despite the mechanical events observed, the biological and esthetic outcomes were favorable. Complete papilla fill was recorded in 70% of cases, while midfacial mucosal recession, bleeding on probing, and suppuration, were each recorded in only 5% of cases. Mean total marginal bone-level change at 1 year was limited. These outcomes are consistent with the concept that immediate provisionalization can help preserve or guide peri-implant soft-tissue architecture. Previous studies have shown that provisional restorations can support soft tissues and help preserve the ridge contour after extraction. For example, Yang et al. reported that ovate pontic provisional restorations after extraction supported papillae and soft tissues, with limited midfacial shrinkage after 6 months [37]. Although that study did not involve implant-supported Morse taper provisionals, it reinforces the broader principle that early prosthetic support can influence soft-tissue healing. The observed tissue outcomes should be interpreted as the result of the complete clinical protocol and not as the isolated effect of the non-antirotational connection. Factors such as guided placement, strict case selection, provisional contour design, insertion torque, occlusal control, Morse taper connection, grafting, socket characteristics, and operator experience may all have contributed to the positive buccal contour gain and low recession rate. Therefore, the suggested benefit of the non-antirotational component remains mechanistic and hypothesis-generating, not causally demonstrated by the present single-arm study.

The present results are also in line with recent digital workflow studies. Donker et al. reported that a digital workflow allowed the manufacture and placement of prefabricated temporary restorations after immediate implant placement, with favorable 1-year clinical, esthetic, radiographic, and patient-reported outcomes [10]. The current study differs by using a non-antirotational provisional, which may provide additional clinical flexibility when the exact rotational position of the implant differs slightly from the planned position. This feature represents a practical advantage, especially when the provisional crown is fabricated before surgery and must be adapted immediately after implant placement.

Occlusal management remains essential. The literature generally recommends nonfunctional provisionalization during early healing to reduce micromotion and the risk of overload. The systematic review by Wittneben et al. included studies with different provisional occlusal schemes but confirmed that immediate restoration/loading can be predictable when case selection and protocols are appropriate [4]. In the present study, occlusion was checked and adjusted when necessary, and no significant association was found between occlusal scheme and dislodgement; however, the sample size was too small to draw strong conclusions.

The proposed protocol can be situated within a continuum of strategies for esthetic-zone single-implant rehabilitation. At one end, ridge preservation using ovate-pontic provisional restorations followed by delayed implant placement [37] may help maintain the soft-tissue contour, but it prolongs the overall treatment timeline by approximately 4–6 months and usually requires an additional surgical phase. At the other end, immediate implant placement combined with immediate provisionalization and simultaneous soft-tissue augmentation—using either a subepithelial connective tissue graft (SCTG) or a xenogeneic collagen matrix (XCM)—has been associated with reduced midfacial mucosal recession and improved interproximal soft-tissue scores [3]. In the present cohort, peri-implant papilla architecture was assessed using the Jemt papilla index [25], and complete papilla fill (Jemt score = 3) was recorded in 70% of cases, with the remaining 30% scoring 2; no case scored ≤1, which is consistent with adequate maintenance of the interproximal soft-tissue contour during the conditioning phase. However, these approaches may involve greater surgical complexity, increased chair time, and additional morbidity, particularly when an autogenous donor site is required for SCTG harvesting. In this context, the present protocol aims to integrate osseointegration and peri-implant soft-tissue modeling within the same healing interval, using the provisional restoration to support and guide mucosal maturation while still allowing soft-tissue grafting to be performed when clinically indicated.

The Marginal Migration Concept of Valavanis et al. [6] emphasizes a flapless, supra-periosteal pouch design with a slowly resorbable membrane and bone substitute, combined with a supragingival, 90-degree-emergence provisional, and reports favorable soft-tissue outcomes even in compromised type II sockets with Miller class I recession defects [6]. Our protocol is most closely aligned with the latter philosophy in that it uses a supragingival provisional contour and avoids any compressive emergence on the marginal mucosa, but differs in two respects: it does not require a customized soft-tissue grafting step in every case, and it transfers the precision of the prosthetic seating from intra-operative chairside relining to a prefabricated, digitally planned crown supported by a non-antirotational conical interface.

This study should be interpreted in light of several limitations. First, the retrospective single-arm design does not allow direct comparison with indexed titanium-base provisionals or conventional immediate provisionalization protocols. Therefore, the findings should be considered preliminary feasibility data from a selected retrospective case-series experience, not evidence of superiority or predictable clinical performance across all esthetic-zone implant indications. Second, the cohort was small and clinically imbalanced. Only 20 cases were included, with a predominance of female patients, anterior implant sites, flapless procedures, and cases receiving soft- and/or hard-tissue grafting. These characteristics limit the generalizability of the findings to broader patient populations, posterior sites, not grafted cases, and different surgical or prosthetic protocols. In addition, the low number of provisional dislodgement events precluded a robust analysis of potential predictors. Third, all procedures were performed by a single experienced implantologist using a highly standardized protocol. While this may have reduced clinical variability, it may also limit transferability to other clinicians, implant systems, laboratories, and digital workflows. Fourth, the specific contribution of the 5-degree Morse taper connection and potential frictional-locking effect cannot be separated from other clinically relevant variables, including implant position, insertion torque, occlusal control, provisional design, grafting, and patient compliance. Finally, the follow-up was limited to 1 year; therefore, longer-term studies are needed to evaluate the stability of the conditioned peri-implant soft-tissue architecture after delivery of the definitive restoration.

Clinical relevance: In selected esthetic-zone single-implant cases, a digitally planned and guide-delivered prefabricated provisional restoration may help streamline the immediate provisionalization workflow and support peri-implant soft-tissue conditioning during healing. The use of a non-antirotational provisional component may provide intraoperative rotational flexibility when minor discrepancies exist between the planned and achieved implant position. Nevertheless, the present findings indicate that this approach should be considered a technique-sensitive protocol requiring adequate primary implant stability, careful verification of provisional seating, nonfunctional occlusal adjustment, patient compliance, and close postoperative monitoring, particularly because provisional dislodgement may occur and require chairside management.

The present study provides preliminary feasibility data and should be followed by prospective controlled studies with larger and more balanced cohorts. Future investigations should compare non-antirotational and indexed provisional components under standardized clinical conditions, quantify the degree of intraoperative rotational adaptation, and measure the relationship between planned and achieved implant position and provisional seating accuracy. Additional studies should also evaluate the reproducibility of this workflow across different clinicians, implant systems, intraoral scanners, surgical-guide manufacturing protocols, and dental laboratories. Longer-term follow-up is required to determine whether the conditioned emergence profile, buccal contour gain, papilla fill, marginal bone stability, and complication rates remain stable after definitive restoration.

## 5. Conclusions

Within the limitations of this retrospective single-arm cohort study, digitally planned and guide-delivered immediate provisionalization using prefabricated non-antirotational provisional crowns on 5-degree Morse taper implants appeared clinically feasible for selected single-implant cases in the esthetic zone. Provisional survival without major intervention was achieved in 75% of cases, while 25% of provisionals presented dislodgement requiring chairside management. Biologic complications were uncommon, complete papilla fill was observed in 70% of cases, mean marginal bone-level change at one year was limited, and all cases showed positive buccal contour change during the tissue-conditioning phase.

These findings should be interpreted as preliminary feasibility data for the complete clinical and digital protocol, not as evidence of superiority over indexed provisional components. Careful case selection, accurate seating, occlusal control, patient compliance, and close follow-up remain necessary. Controlled studies with larger samples and comparator groups are required to determine whether the non-antirotational design provides measurable advantages over conventional indexed provisionalization.

## Figures and Tables

**Table 1:** Inclusion and exclusion criteria.

Inclusion Criteria	Exclusion Criteria
Single implant placed in the esthetic zone	Multiple-unit implant restorations
Guided implant placement performed	Splinted provisional restorations
Same-day immediate provisionalization	Full-arch implant rehabilitations
Prefabricated provisional crown designed according to the planned implant position and emergence profile	Absence of key clinical records
Use of a non-hexagonal/non-antirotational provisional component	Absence of essential digital planning files or intraoral scan records
Screw-retained provisional restoration connected to a 5-degree Morse taper implant	Absence of radiographic follow-up documentation
Adequate primary implant stability allowing immediate provisionalization	Absence of follow-up documentation
At least 1 year of follow-up after definitive crown delivery	Documented uncontrolled parafunction or other conditions that could compromise provisional stability or implant rehabilitation

The term “esthetic zone” referred to single-implant sites located in the anterior maxilla and visible premolar region where peri-implant soft-tissue architecture and emergence-profile development were considered clinically relevant.

**Table 2:** Patient-, site-, and surgery-level characteristics of the 20 cases.

Variable	Statistic/Category	Value (*n* = 20)
Age (years)	Mean ± SD	38.1 ± 12.7
	Median [IQR]	36 [26.75–45.25]
	Range	25–67
Gender	Female	19/20 (95.0%)
	Male	1/20 (5.0%)
Smoking status	Non-smoker	14/20 (70.0%)
	Smoker	6/20 (30.0%)
Periodontal history	Gingivitis	13/20 (65.0%)
	Chronic periodontitis	6/20 (30.0%)
	None/not reported	1/20 (5.0%)
Systemic conditions	None	18/20 (90.0%)
	Hypertension (HTA)	2/20 (10.0%)
Bruxism/parafunction	No	20/20 (100%)
Tooth position	Anterior (11–13, 21–23)	18/20 (90.0%)
	Premolar (14, 25)	2/20 (10.0%)
Tissue phenotype	Thin	12/20 (60.0%)
	Thick	4/20 (20.0%)
	Mixed	4/20 (20.0%)
Socket status	Healed	13/20 (65.0%)
	Post-extraction (immediate)	7/20 (35.0%)
Flapless approach	Yes	19/20 (95.0%)
Grafting	Soft tissue only	10/20 (50.0%)
	Soft + hard tissue	8/20 (40.0%)
	None	2/20 (10.0%)
Implant diameter (mm)	Mean ± SD	3.72 ± 0.26
	3.5 mm/4.0 mm	11 (55.0%)/9 (45.0%)
Implant length (mm)	Mean ± SD (range)	11.8 ± 1.0 (10–13)
Insertion torque (N·cm)	Mean ± SD	34.75 ± 2.55
	30/35/40 N·cm	3 (15.0%)/15 (75.0%)/2 (10.0%)
Occlusal scheme	Anterior guidance	15/20 (75.0%)
	Group guidance	4/20 (20.0%)
	Canine guidance	1/20 (5.0%)

**Table 3:** Mechanical, biologic, esthetic, and process-related secondary outcomes.

Outcome	n/N	%	Wilson 95% CI
Chairside adjustment at delivery (any)	1/20	5.0%	0.9–23.6%
Midfacial mucosal recession (1 mm)	1/20	5.0%	0.9–23.6%
Complete papilla (papilla score = 3)	14/20	70.0%	48.1–85.5%
Bleeding on probing	1/20	5.0%	0.9–23.6%
Suppuration	1/20	5.0%	0.9–23.6%
Any unplanned visit (>0 visits)	8/20	40.0%	21.9–61.3%

n = number of events; N = total number of included cases. Number of unplanned visits (continuous): mean 1.00 ± 1.38; median 0 [IQR 0–2]; range 0–4.

**Table 4:** Marginal bone-level change (mm) measured on OPTs (*n* = 20).

Time Interval	Mean ± SD	Median [IQR]	Range	<0.5 mm	<1.0 mm
Early (T0 → T3)	0.24 ± 0.20	0.20 [0.08–0.33]	0.02–0.84	19/20 (95.0%)	20/20 (100%)
Late (T3 → T4)	0.14 ± 0.03	0.13 [0.12–0.16]	0.10–0.18	20/20 (100%)	20/20 (100%)
Total (T0 → T4)	0.37 ± 0.20	0.33 [0.22–0.48]	0.14–0.96	16/20 (80.0%)	20/20 (100%)

**Table 5:** Buccal contour change (T0 vs. T2, intraoral scan superimposition) overall and by subgroup.

Variable/Subgroup	*n*	Mean ± SD (mm)	Median [IQR] (mm)
Overall cohort	20	1.41 ± 0.48	1.44 [1.05–1.71]
Socket—healed	13	1.45 ± 0.40	1.57 [1.28–1.69]
Socket—post-extraction	7	1.33 ± 0.63	1.10 [0.90–1.71]
Phenotype—Thin	12	1.42 ± 0.41	1.54 [1.10–1.69]
Phenotype—Mixed	4	1.60 ± 0.76	1.71 [1.12–2.19]
Phenotype—Thick	4	1.19 ± 0.38	1.09 [0.90–1.38]
Grafting—Soft tissue alone	10	1.51 ± 0.39	1.58 [1.40–1.74]
Grafting—Soft + hard tissue	8	1.26 ± 0.62	1.01 [0.85–1.49]
Grafting—None	2	1.48 ± 0.29	1.48 [1.38–1.59]

Subgroup comparisons were exploratory and did not reach statistical significance: Mann–Whitney U for healed vs. post-extraction sockets *p* = 0.500; Kruskal–Wallis across phenotypes *p* = 0.651. The buccal contour gain was numerically largest in mixed-phenotype sites and smallest in thick-phenotype sites, but the small sample size and the imbalance across categories preclude any inferential conclusion.

**Table 6:** Exploratory associations between continuous outcomes and provisional dislodgement (Mann–Whitney U).

Variable	Dislodged (*n* = 5)—Median [IQR]	Retained (*n* = 15)—Median [IQR]	U	*p*
Number of unplanned visits	2 [2–4]	0 [0–1]	61.5	0.019
Papilla score	3 [3–3]	3 [2–3]	52.5	0.111
Age (years)	33 [27–35]	38 [26.5–46]	23.0	0.220
Insertion torque (N·cm)	35 [35–35]	35 [35–35]	40.0	0.818
Early bone loss (mm)	0.33 [0.19–0.33]	0.20 [0.08–0.32]	47.5	0.406
Late bone loss (mm)	0.13 [0.12–0.14]	0.13 [0.12–0.17]	37.5	1.000
Total bone loss (mm)	0.47 [0.32–0.49]	0.33 [0.22–0.46]	47.5	0.407
Buccal contour gain (mm)	1.28 [1.27–1.66]	1.51 [0.99–1.73]	41.0	0.793

## Data Availability

The data collected in this study are available from the corresponding authors upon reasonable request.

## Supplementary Materials

The following supporting information can be downloaded at: https://www.mdpi.com/article/10.3390/jcm15103945/s1, The STROBE checklist for reporting observational studies is provided as Supplementary Materials.

## Abbreviations

The following abbreviations are used in this manuscript:

STROBE	Strengthening the Reporting of Observational Studies in Epidemiology statement for observational studies
OPT	Orthopantomogram
IOS	Intraoral scan
CBCT	Cone Beam Computed Tomography
SCTG	Subepithelial connective tissue graft
XCM	Xenogeneic collagen matrix
RMS	Root mean square
ALARA	As Low As Reasonably Achievable
